# A DSC Test for the Early Detection of Neoplastic Gastric Lesions in a Medium-Risk Gastric Cancer Area

**DOI:** 10.3390/ijms24043290

**Published:** 2023-02-07

**Authors:** Valli De Re, Stefano Realdon, Roberto Vettori, Alice Zaramella, Stefania Maiero, Ombretta Repetto, Vincenzo Canzonieri, Agostino Steffan, Renato Cannizzaro

**Affiliations:** 1 Immunopathology and Cancer Biomarkers, Centro di Riferimento Oncologico di Aviano, CRO Aviano, National Cancer Institute, IRCCS, 33081 Aviano, Italy; 2 Oncological Gastroenterology, Centro di Riferimento Oncologico di Aviano (CRO) IRCCS, 33081 Aviano, Italy; 3 Gastroenterology Unit, Veneto Institute of Oncology IOV-IRCCS, Via Gattamelata 64, 35128 Padua, Italy; 4 Department of Surgery, Oncology, and Gastroenterology (DiSCOG), University of Padua, Via Giustiniani 2, 35128 Padua, Italy; 5 Pathology Unit, Centro di Riferimento Oncologico di Aviano (CRO) IRCCS, 33081 Aviano, Italy; 6 Department of Medical, Surgical and Health Sciences, University of Trieste, 34127 Trieste, Italy

**Keywords:** gastric cancer, pepsinogen, gastrin G17, *Helicobacter pylori*, screening

## Abstract

In this study, we aimed to assess the accuracy of the proposed novel, noninvasive serum DSC test in predicting the risk of gastric cancer before the use of upper endoscopy. To validate the DSC test, we enrolled two series of individuals living in Veneto and Friuli-Venezia Giulia, Italy (n = 53 and n = 113, respectively), who were referred for an endoscopy. The classification used for the DSC test to predict gastric cancer risk combines the coefficient of the patient’s age and sex and serum pepsinogen I and II, gastrin 17, and anti-*Helicobacter pylori* immunoglobulin G concentrations in two equations: Y1 and Y2. The coefficient of variables and the Y1 and Y2 cutoff points (>0.385 and >0.294, respectively) were extrapolated using regression analysis and an ROC curve analysis of two retrospective datasets (300 cases for the Y1 equation and 200 cases for the Y2 equation). The first dataset included individuals with autoimmune atrophic gastritis and first-degree relatives with gastric cancer; the second dataset included blood donors. Demographic data were collected; serum pepsinogen, gastrin G17, and anti-*Helicobacter pylori* IgG concentrations were assayed using an automatic Maglumi system. Gastroscopies were performed by gastroenterologists using an Olympus video endoscope with detailed photographic documentation during examinations. Biopsies were taken at five standardized mucosa sites and were assessed by a pathologist for diagnosis. The accuracy of the DSC test in predicting neoplastic gastric lesions was estimated to be 74.657% (65%CI; 67.333% to 81.079%). The DSC test was found to be a useful, noninvasive, and simple approach to predicting gastric cancer risk in a population with a medium risk of developing gastric cancer.

## 1. Introduction

Gastroscopy is the standard procedure for gastric cancer (GC) diagnosis with a false-negative rate of about 19% [1]. This procedure is invasive, time-consuming, and uncomfortable.

The 5-year GC survival rate is poor, reaching approximately 30% in Europe [2]. Studies in countries such as Japan and Korea have shown a high incidence of GC, with a 30% reduction in GC mortality due to gastroscopy screening programs [3].

Overall, a GC diagnosis at an early/asymptomatic stage not only improves clinical outcomes [4] but is associated with endoscopic resections in most cases, resulting in the now-standard treatment for early gastrointestinal cancers without regional lymph node metastasis [5].

A population with an age-standardized incidence rate (ASIR) of 10 to 20 per 100,000 is considered to have an intermediate risk of developing GC. According to published data, in northern Italy, the ASIR was calculated as 12 to <14 in 2017, on average twofold higher in males (33.9) than in females (17.0) [6,7]; thus, Italy is a geographic area whose population has an intermediate risk of developing GC.

At present, the screening for GC is only performed in countries with an elevated risk of GC, such as Japan and Korea [8]. In a country with an intermediate risk of GC, using gastroscopy as first-line testing alone is not considered feasible due to its invasiveness and expensive cost [9]. On the other hand, the inappropriateness of upper endoscopies leads to decreased diagnostic yield [10,11]. Thus, it is necessary to consider a less invasive and more cost-effective solution to find subjects at risk of developing GC. Moreover, it is necessary to take changes in GC epidemiology that have occurred over the last few decades into consideration. GC epidemiology has changed concomitantly with a reduction in *Helicobacter pylori *(*H. pylori*)** infections, but also with an increased incidence of cardia GC and an increase in GC diagnoses and mortality in younger adults [12].

The available pepsinogen (PG) test (PG test) is based on a combination of the serum PG-I level and the PG-I/PG-II ratio, which is used as a marker for chronic atrophic gastritis (CAG), and it has been widely proposed for the selection of patients at risk for GC [13,14].

However, while the PG test is accurate for CAG diagnosis, it suffers from an unsatisfactory specificity in predicting GC due to using the widely standardized cutoff points of PGI < 70 ng/mL and PGI/PGII < 3.0 [15,16,17]. Therefore, the ability to predict GC risk needs to be improved.

Recently, the addition of serum gastrin G17 (G17) and anti-*H. Pylori* immunoglobulin (IgG) to the PG test was proposed to improve the diagnosis of atrophic gastritis and GC [18]. PGI is only secreted by the fundic glands; PGII is secreted by the fundic glands, the pylorus, and the Brunner glands; and gastrin G-17 is only secreted by gastric antral G cells. Accordingly, serum PG and G17 levels can be used to localize morphology and detect the extension of gastric lesions [19]. Gastrin G17 might also sustain the proliferation and migration of epithelial gastric cells [20]. *H. pylori* is pathogenetic for GC and a subtype of autoimmune gastritis [21]. Age ≥ 60 and male sex have been reported to be independent risk factors for GC in several studies [22,23].

Based on our earlier study [24] and two new datasets in this work, first (the discovery part), we developed a model—herein called the DeRe–Steffan–Cannizzaro (DSC) test—to discriminate patients at risk of developing GC. Briefly, the model is based on regression analyses to calculate the coefficient of the patient’s age and sex and their PG, anti-*H. Pylori* immunoglobulin (IgG), and serum gastrin G17 levels.

Second (the validation part), we validated the DSC test. Two monocenter studies were conducted involving 53 retrospectively selected (based on their diagnosis) individuals who presented at the Gastroenterology Unit of the Veneto Institute of Oncology IOV-IRCCS, Padua, Italy (validation cohort 1), and 113 consecutive individuals who were referred by physicians to the oncological gastroenterology unit of the Centro di Riferimento Oncologico of Aviano, Italy (validation cohort 2). Physician indications included dyspepsia, preneoplastic lesions, and/or suspected GC. Dyspepsia was defined as upper gastrointestinal symptoms, such as gastric pain and/or burning, without a typical disease and with no clear cause [10,11]. The preneoplastic lesions were diagnosed via gastroscopy and histologically examining the biopsies [25,26,27,28,29]. The GC diagnoses were confirmed via histopathological examinations [30,31]. Data on each patient’s age and sex were collected, and expression profiles of serum PG, gastrin G17, and *H. Pylori* IgG were performed in order to group patients based on GC risk classes into negative, neutral, and positive results, respectively. The accuracy of the model was then demonstrated with gastroendoscopy and a histological diagnosis.

This is the first study on GC risk prediction adopting the DSC model. The model showed an overall 74.66% accuracy rate for GC diagnosis, notably improving the detection sensitivity from 15.00% to 70.00% and retaining a good specificity (74.66%) compared with the standard cutoff for PG tests. This study could help physicians make better decisions and allocate proper resources to improve GC prevention, for example, by removing high-grade preneoplastic lesions and selecting patients for endoscopic surveillance. In addition, by diagnosing opportunistic GC at an early stage, the GC survival rate can be improved [2,3].

## 2. Results

### 2.1. Study Design

**Figure 1:**
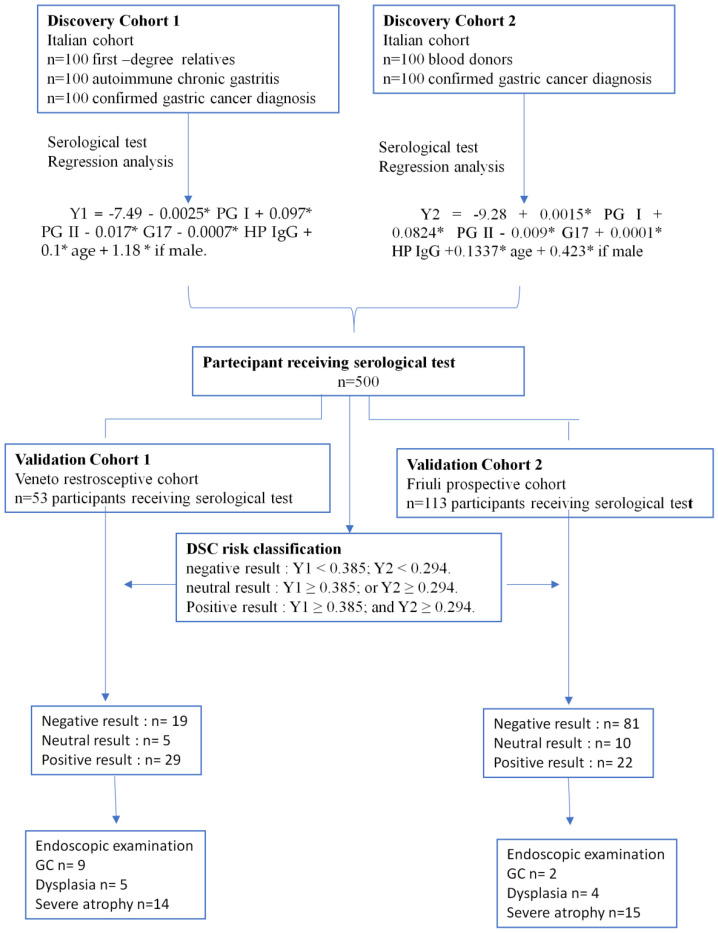
Schematic of the study design and main results.

Based on our previous observations [24] and the two new cohorts in this work (Figure 1, discovery cohort 1 and discovery cohort 2), we developed a model based on the coefficients of the patient’s age, sex, and serum PG, *H. Pylori* IgG, and gastrin G17 levels to discriminate patients at an elevated risk of GC. The results of the Y1 and Y2 equations obtained from discovery cohort 1 and discovery cohort 2 are reported in Figure 1. Data from all the study’s participants are reported in Table 1. The median G17 level in the female category in discovery cohort 1 was higher than in the other groups due to the prevalence of females affected by autoimmune atrophic gastritis [24]. In discovery and validation cohorts, *H. pylori* IgG and PG II levels were higher in individuals >65 years old, in accordance with the decreased incidence of *H. pylori* infection in the global population [12] and increases in serum PG II levels often found in individuals infected with *H. pylori* [24].

Next, we recruited two validation cohorts, the first consisting of a retrospective series of selected participants from the Veneto region (validation cohort 1, n = 53) and the second consisting of a prospective series of consecutive participants who were referred to gastroenterologists for gastroscopy from the Friuli geographic area (validation cohort 2, n = 113). The study participants’ data are reported in Table 1.

### 2.2. DSC Classifications for GC Risk

Two validation cohorts were enrolled in the study. The first cohort consisted of a retrospective series of 53 participants (validation cohort 1); the second cohort consisted of a prospective series of 113 participants consecutively enrolled since May 2020 (validation cohort 2).

The DSC test measures the serum biomarkers and demographic data of the participants. The patients’ data are reported in Table 1. Based on the results of both the DSC Y1 and Y2 equations, Y1 < 0.385 and Y2 < 0.294 (as reported in Figure 1 and described in the methods section), we classified participants receiving the serological test as having negative, neutral, or positive DSC results.

The results of the DSC test are reported in Table 2 according to the calculated Y1 and Y2 equations for each participant. Based on these criteria, 19 (35.8%) and 81 (71.7%) participants were classified as negative; 5 (9.4%) and 10 (8.8%) were classified as neutral; and 29 (54.7%) and 22 (19.5%) were classified as positive for GC risk in validation cohort 1 and validation cohort 2, respectively.

### 2.3. Gastroscopy and Histopathological Diagnosis

The same participants were classified after using gastroscopy and histological examinations to diagnose them with GC, dysplasia, severe atrophy (OLGA stages III–IV), or no/moderate-grade atrophy (OLGA stages 0–II). The diagnosis results are summarized in Table 3.

In the first cohort, consisting of a retrospective series of participants (validation cohort 1), the diagnosis was GC in nine patients, dysplasia in five, and severe atrophy in 14. The remaining participants (n = 25) showed no atrophy or mild–moderate atrophy (OLGA stages 0–II).

Of the 113 consecutive participants in the second cohort (validation cohort 2), a neoplastic lesion was diagnosed in two patients (i.e., one advanced, differentiated, diffuse-type GC with middle tumor infiltration; one early antrum GC), dysplasia was diagnosed in three patients (one adenoma with high-grade dysplasia in the corpus; one low-grade dysplasia with intestinal metaplasia and neuroendocrine hyperplasia in the antrum; one dysplasia with intestinal metaplasia in the corpus), a preneoplastic adenoma was diagnosed in one case, and severe atrophy was diagnosed in 15 cases; the remaining participants (n = 92) showed no atrophy or mild–moderate atrophy (OLGA stages 0–II).

In the patient with an early GC and the four patients with dysplasia, mini-invasive gastric resections were conducted via endoscopy.

### 2.4. DSC Classification Accuracy

A comparison between the DSC classification results and a histological classification was used to calculate the accuracy of the DSC test. A subgroup analysis was performed separately for validation cohort 1 and validation cohort 2, and then for all cases.

In both validation cohorts, we observed a substantially larger risk of GC in individuals classified as DSC-positive (validation cohort 1: 7/9 (77.78%); validation cohort 2: 2/2 (100%)) than those classified as DSC-neutral (validation cohort 1: 1/5 (20.0%); validation cohort 2: 0/10) or DSC-negative (validation cohort 1: 1/19 (5.3%); validation cohort 2: 0/81). The relative risk of GC diagnosis in individuals classified with a positive DSC score was RR 2.90 (95%CI; 0.67–12.67) and RR 20 (95%CI; 0.99–402.45) in validation cohort 1 and validation cohort 2, respectively.

**Figure 2:**
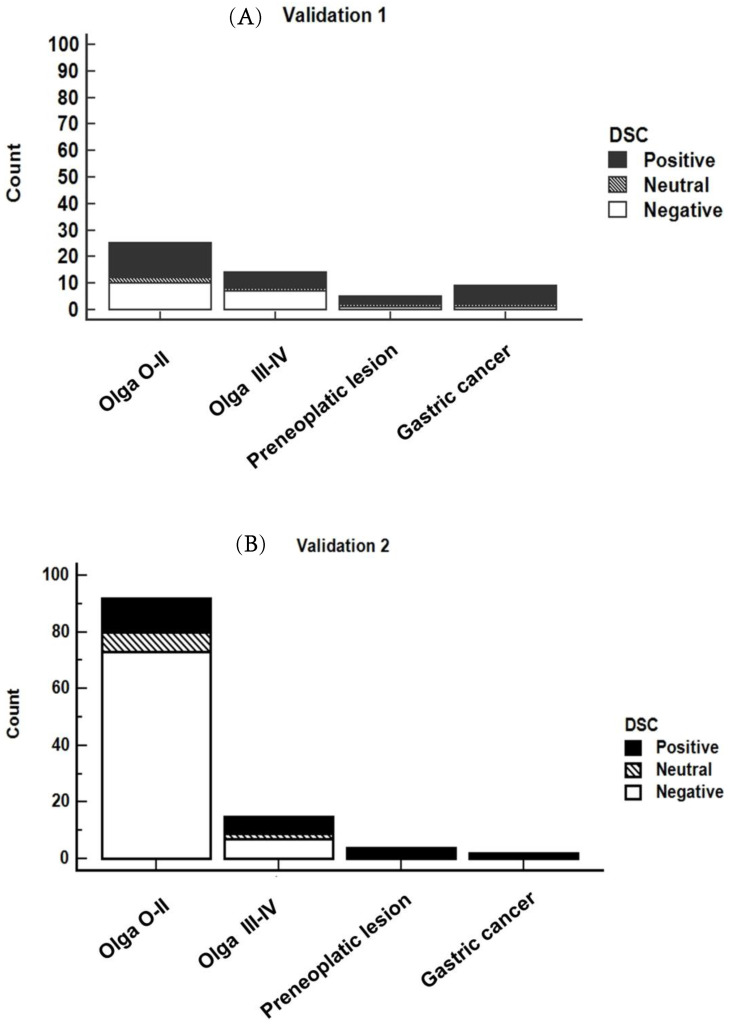
Distribution of DSC results taken from the validation 1 (**A**) and validation 2 (**B**) datasets according to the diagnostic categories.

Figure 2 shows the distribution of DSC-positive, -neutral, and -negative results in the validation datasets according to the individual diagnosis of each participant.

Based on the DSC test, 60.2% of the overall participants in the validation cohorts were classified as negative, 9.0% as neutral, and 30.7% as positive for GC risk (Table 2).

The predictive value of the DSC test for the risk of GC was calculated using a diagnostic test; the AUC value was 0.723 considering all participants in the validation datasets (0.614 in validation cohort 1 and 0.749 in validation cohort 2). The overall accuracy was 74.657%, with a calculated disease prevalence of about 0.01% in the general Italian population [6] (see details in Table 4).

### 2.5. Reproducibility of the DSC Method

In 26 participants (17 negative, three neutral, and six positives in the DSC test), the test was repeated after a median interval of 15.5 months (IQR, 10 to 19 months) (Table 5). We found a change in DSC classifications from negative to neutral in two participants with a diagnosis of mild–moderate atrophy (OLGA 0-II category). In the remaining 24 cases, both the classification and the diagnosis remained the same (Table 5).

### 2.6. Comparison of the Overall Validation Process (n = 166 Cases) Using the DSC Test and the Standardized Pepsinogen Test

The standard cutoff for the PG test is PG I < 70 ng/mL and a PG I/PG II ratio of ≤3.0, resulting in an area under the curve (AUC) of 0.470 (Table 6). For the DSC test, the AUC was 0.723, a better AUC, mostly due to the increase in the sensitivity value from 15.00% to 70.00%. Because the predictive value depends on the prevalence of the disease (0.01% in the general population of Italy), the predictive value for positive cases was 0.03% when using the DSC test and 0.01% when using the PG test, while the overall accuracy remained similar between tests.

## 3. Discussion

In this work, we proposed a screening strategy to select individuals at risk for GC based on our newly developed DSC test. The DSC test showed good accuracy (74.66%) with an increase in sensitivity when compared with the PG test, meaning that it could be helpful in identifying gastroenterological patients for opportunistic GC screening in medium-risk areas, e.g., Italy. The DSC test is noninvasive, reproducible, and has high specificity (i.e., a true negative rate). In individuals with a positive DSC classification, gastroscopy and surveillance should be recommended to detect GC at an early stage and improve prevention rates, for example, by using mini-invasive cures.

Earlier studies have confirmed the high risk of GC in patients with atrophic and/or metaplastic gastritis. In particular, two pathological classifications developed from the Sydney System to grade long-standing gastritis and metaplasia (the OLGA and OLGIM classifications) have been shown to be informative in determining severe atrophy/metaplasia (stage III-IV) and an increased risk of GC development [32,33,34]. Based on these results, gastroenterology guidelines recommend an endoscopic follow-up every 3 years in individuals with a diagnosis of severe atrophy/metaplasia [35]. Performing the DSC test on these patients could be a useful approach for clinical practices better detect individuals at higher risk, who would then be examined using a stricter endoscopic approach in the follow-up.

The results obtained from using the DSC test on the prospective, non-selected, individual cohort (validation cohort 2) are comparable to those achieved using a similar approach in an area of high GC incidence (GC prevalence, 2.84%; ROC curve predicting GC, 0.79). We propose using this test as an opportunistic screening method in individuals attending the gastroenterology attention to improve the detection rate of GC at an early stage [36].

To the best of our knowledge, there has only been one prospective study that combined the pepsinogen test in a population with a medium GC risk [37], but the study was focused on surveilling patients with precancerous lesions (atrophic gastritis, intestinal metaplasia, dysplasia). The authors showed that only patients with extended precancerous lesions with a low PGI/II ≤ 3 ratio and/or OGLIM stage (III-IV) developed high-grade dysplasia or neoplasia at follow-ups after about 57 months. However, it is noteworthy that atrophy is usually associated with *H. pylori* infection and intestinal-type GC. The proposed DSC model introduces the possibility of selecting individuals at high risk of opportunistic neoplastic lesions for further endoscopic examinations. In our study, at a median follow-up of 15.5 months, two individuals out of 17 showed an increase in DSC classification from the negative category to the neutral category, however the histological diagnosis remained at moderate atrophy (OLGA stage 0-II).

GC is a disease of old age, with about one-half of patients with GC being over age 65. A primary characteristic of aging is a progressive loss of physiological gastric tissue integrity that, in turn, leads to impaired function and increased alteration of the PG-I/PG-II ratio, which leads to a decline in gastric acidity [37] and a low circulating concentration of vitamin B12 [38,39]. This could justify a potential decrease in the accuracy of the DSC classification in older subjects (>75 years old); as such, it is necessary to take this aspect into consideration regarding the idea of screening the general population. Therefore, it may be helpful to add other biomarkers to increase the DSC accuracy by reducing the number of false positives in particular in individuals who are >75 years old.

Several studies have reported the serum indicators of GC. Some of them have been proposed in combination with pepsinogen to improve the accurate diagnosis of GC, e.g., sugar carbohydrate antigen 72-4 (CA72-4) [40], CEA, CA12-5, and CA19-9 [41]; metabolites such as hydroxylated sphingomyelins (SM(OH)) and acylcarnitine derivatives (C2, C16, and C18:1) [42]; alcohol dehydrogenase (ADH) activity [43]; interleukin-6 (IL-6); human epididymal protein 4 (HE-4); adiponectin; ferritin and Krebs von den Lungen (KL-6) [44]; soluble T cell immunoglobulin; and mucin domain molecule 3 (sTim-3) [45]. Overall, these results are interesting, but they are all preliminary and need to be evaluated in large prospective studies in combination with the DSC test.

In the last few years, new, increasingly complex technological strategies have emerged, such as the single-cell RNA sequencing (scRNA) transcriptome, which can characterize cellular and molecular networks present in a tissue at the same time [46]. scRNA offers the possibility of finding new GC diagnostic markers by reducing the complexity of gene RNA expression patterns in distinct cell populations. This approach is also useful in deciphering GC pathogenesis and detecting rare, tumor-specific cells at the onset of early GC stages. Currently, only one study has reported potential diagnostic markers for GC using this technology [46], but candidates have also been found in other tissues. Moreover, it is necessary to screen candidates such as secretory proteins, which are abundantly expressed in GC cells and could be evaluated in the serum alone or in addition to the DSC test for GC prediction. Furthermore, their sensitivity and specificity in comparison to the DSC test should be assessed.

Our study had limitations due to the limited number of cases tested and its application in a unique laboratory. Its use in other medium-risk GC populations is necessary to support its validity.

## 4. Materials and Methods

### 4.1. Study Cohorts

This study was based on four cohorts of participants: two discovery cohorts (discovery cohort 1 and discovery cohort 2) and two validation cohorts (validation cohort 1 and validation cohort 2). Demographic and serological data were collected from all participants. Subjects who initially declined consent were excluded from the study.

Discovery cohort 1 included 300 retrospectively selected subjects at risk of GC, i.e., subjects with a family history of GC, autoimmune chronic atrophy gastritis, severe precancerous lesions, or a diagnosis of GC.

Discovery cohort 2 included 200 subjects: blood donors and patients with a GC diagnosis.

Validation cohort 1 consisted of 53 selected individuals from a retrospective series of cases with no atrophy, mild–moderate atrophy, dysplasia, or GC enrolled in the Veneto region, Italy.

Validation cohort 2 consisted of prospective participants recruited from May 2020 to September 2022 (n = 113). Cases were consecutive participants who were referred to a gastroenterologist by a physician for gastroscopy.

Participants from the four cohorts received serological tests to determine their serum PG, G17, and *H. pylori* IgG values. Participant data according to the cohorts are detailed in Table 1.

The study was conducted following the Declaration of Helsinki and approved by the Unique Regional Ethics Committee for Friuli-Venezia Giulia (approval number CRO-2019-46). Written informed consent was obtained from all participants.

### 4.2. Construction of the DSC Model

Data from each participant enrolled in discovery cohort 1 and discovery cohort 2 were used to perform a regression analysis that discriminated patients with GC and determined the coefficients of each variable (i.e., PG, age, sex, *H. pylori* IgG, and gastrin G17)

The resulting Y1 and Y2 equations discriminated patients affected by GC in discovery cohort 1 and discovery cohort 2, respectively.

Y1 = −7.49 − 0.0025 × PG I + 0.097 × PG II − 0.017 × G17 − 0.0007 × HP IgG + 0.1 × age + 1.18 × if male.

Y2 = −9.28 + 0.0015 × PG I + 0.0824 × PG II − 0.009 × G17 + 0.0001 × HP IgG +0.1337 × age + 0.423 × if male.

To establish the best cutoff for the discrimination, we used an ROC curve analysis.

The combination of the Y1 and Y2 results, based on cutoffs of Y1 < 0.385 and Y2 < 0.294, determined the DSC classification model.

In detail, subjects positive for Y1 > 0.385 and Y2 > 0.294 were classified as having high GC risk; subjects positive for only one (Y1 or Y2) were classified as having medium risk; and subjects were deemed low risk when any of the Y1 or Y2 criteria were satisfactory.

### 4.3. DSC Model Assessment in the Validation Cohorts

Participants in the validation cohorts were classified using the DSC model. The DSC classification of each participant was recorded in a database. For each participant, the data were correlated with the diagnosis obtained via gastroscopy and the histological examination of the biopsies.

### 4.4. Serological Data

Blood samples of approximately 5 mL each were obtained from all participants after 10 h of fasting. The tubes were centrifugated for 10 min at ≥10,000 rpm. The serum was stored immediately at −20 °C until an assay was performed. Serologic testing for *H. pylori*-IgG, PGI, PGII, and gastrin G17 was performed using an automated chemiluminescence immunoassay (CLIA) Maglumi analyzer (Medical Systems). Recommended cutoff points, as reported by the manufacturer, were PGI: 70–240 ng/mL, PGII: < 13 ng/mL, G17: 2–10 pmol/L, H.p IgG titer: <30 EIU. A combination of PGI < 70 ng/mL and a PGI/PGII ratio of <3 is informative for atrophic gastritis (AG).

### 4.5. Diagnosis

A gastroendoscopy was performed by gastroenterologists on all participants, and biopsies were collected (two biopsies from the antrum, two from the body, and one from the incisure). Gastric mucosae were examined with high-definition (HD) white-light endoscopy and narrow-band imaging (NBI) to improve the visibility of blood vessels and mucosal structures.

Pathological examinations of biopsy samples fixed in buffered formalin (10%) were conducted by an expert pathologist, and the results were reported according to updated OLGA stages to define gastritis [29], and Lauren [30] and Who [31] for GC classifications of gastric tumors and dysplasia. According to the pathological examinations, participants were classified into three groups: without atrophy or metaplasia, with mild–moderate preneoplastic gastric lesions (atrophy or metaplasia), with dysplasia, and with neoplastic lesions.

### 4.6. Statistical Analyses

All analyses were conducted using MedCalc Statistical Software, version 19.0.4 (MedCalc Software bvba, Ostend, Belgium). The results were summarized using age intervals and descriptive statistics. Comparisons between groups were made using a one-way ANOVA test. The predicted DSC scores for each patient were categorized into low-risk, medium-risk, and high-risk groups based on results of the Y1 and Y2 equations as follows: high-risk: Y1 > 0.385 and Y2 => 0.294; medium-risk: Y1 > 0.385 or Y2 => 0.294; low-risk: neither Y1 > 0.385 nor Y2 => 0.294. Considering the histopathology of the gastric lesions, the subjects were categorized into three groups: without lesions, with mild–moderate gastric lesions (chronic and/or autoimmune atrophic gastritis, intestinal metaplasia), and with severe gastric lesions (dysplasia, early GC, and advanced GC). Categorical data were entered into a two-way table by counting the number of observations that fell into each group of variables. A Spearman’s rank correlation was used to test the relationship between the DSC and histological categories. To test the accuracy of the high-risk DSC model in predicting the diagnosis of dysplasia and GC, we used a diagnostic test; *p* < 0.05 was considered statistically significant.

## 5. Conclusions

We developed the DSC test model and assessed its accuracy for the classification of people at a high risk of developing GC in two validation datasets. The DSC test achieved a good accuracy of 74.66% and a sensitivity increase of 70.00% compared with 15.00% for the PG test, which supports its potential utility in clinical practice for opportunistic GC identification and the selection of patients at elevated risk for strict follow-ups. It remains to be seen whether the DSC test is effective in follow-ups, since gastric lesions may progress or develop later.

## Figures and Tables

**Table 1:** Distribution of demographic and serological data in the discovery and validation cohorts according to sex and age categories.

		Discovery Cohort 1		Discovery Cohort 2		Validation Cohort 1		Validation Cohort 2 n = 113	
		Median (IQ: 25–75%)	Median (IQ: 25–75%)	Median (IQ: 25–75%)	Median (IQ: 25–75%)	Median (IQ: 25–75%)	Median (IQ: 25–75%)	Median (IQ: 25–75%)	Median (IQ: 25–75%)
Biomarkers	Sex	Age ≤ 65	Age > 65	Age ≤ 65	Age > 65	Age ≤ 65	Age > 65	Age ≤ 65	Age > 65
G17	F	13.9 (3.5–50.3)	19.5 (3.4–61.8)	4.0 (1.9–16.3)	17.6 (2.7–27.9)	7.3 (3.9–58.6)	5.3 (3.4–12.4)	5.1 (2.2–52.2)	11.1 (2.7–53.1)
	M	9.5 (3.3–32.9)	15.2 (5.9–38.2)	2.9 (0.5–8.4)	17.1 (7.1–38.3)	8.0 (4.8–36.1)	16.3 (7.9–32.7)	5.0 (2.7–13.9)	31.8 (10.5–119.0)
PGI	F	72 (25.8–119.3)	87.5 (23.7–141.4)	63.6 (49.4–173.0)	127.7 (87.5–210.3)	78.6 (74.5–139.5)	102.9 (56.0–251.2)	60.3 (39.5–105.0)	78.7 (30.8–144.4)
	M	103.2 (70.4–182.9)	76.4 (38.0–150.8)	82.3 (50.5–131.2)	83.7 (38.0–212.8)	119.5 (101.4–198.1)	137.2 (53.2–304.5)	69.7 (35.0–108.7)	73.5 (16.9–263.7)
PGII	F	10.1 (7.1–16.9)	14.0 (8.0–18.1)	9.0 (4.8–19.8)	19.3 (16.0–27.1)	9.9 (7.1–11.2)	13.0 (9.2–20.5)	10.9 (6.2–13.7)	12.7 (10.0–14.8)
	M	13.9 (8.7–21.7)	12.6 (7.5–16.8)	9.0 (6.2–17.0)	13.0 (9.2–20.4)	11.5 (9.6–14.5)	12.9 (9.7–19.7)	9.6 (7.8–12.2)	11.7 (7.9–19.6)
PGI/PGII	F	6.7 (2.3–11.6)	6.6 (3.0–9.9)	8.3 (6.4–10.7)	6.0 (4.9–7.8)	10.1 (7.9–11.9)	9.2 (7.1–10.5)	7.2 (4.3–9.9)	6.8 (3.0–8.4)
	M	8.43 (5.5–12.2)	6.3 (3.0–12.0)	9.2 (6.3–11.6)	6.3 (3.0–12.0)	10.7 (10.3–13.4)	9.1 (7.4–12.5)	7.6 (3.4–8.7)	6.7 (1.8–11.1)
H. pylori IgG	F	14.4 (5.8–72.9)	38.1 (11.5–60.8)	27.0 (4.4–76.4)	47.0 (16.8–71.9)	5.4 (3.6–21.6)	14.5 (3.4–41.2)	5.9 (2.6–16.8)	10.8 (3.7–32.1)
	M	20.6 (6.0–85.6)	61.3 (11.5–111.2)	15.0 (3.85–80.38)	79.4 (21.7–114.1)	3.8 (2.6–12.1)	3.1 (2.6–10.6)	6.4 (3.7–9.2)	15.6 (6.3–35.4)

**Table 2:** DSC GC risk classifications for participants in the validation cohorts.

DSC	Cohorts					
GC Risk Classification	Validation 1		Validation 2		Overall Validation Process	
	n.	%	n.	%	n.	%
Negative	19	35.8	81	71.7	100	60.2
Neutral	5	9.4	10	8.8	15	9.0
Positive	29	54.7	22	19.5	51	30.7
Total	53		113		166	

**Table 3:** Aggregation of participants according to histopathological diagnosis.

Diagnosis	Cohorts			
	Validation Cohort 1 N = 53		Validation Cohort 2 N = 113	
	n. Patients	%	n. Patients	%
Atrophy (OLGA stages 0–II)	25	47.2	92	81.4
Severe atrophy (OLGA stages III–IV)	14	26.4	15	13.3
Dysplasia/preneoplastic lesion	5	9.4	4	3.5
Gastric cancer	9	17.0	2	1.8

**Table 4:** Prediction value of DSC test for GC risk.

	Validation 1 53 Selected Retrospective Cases		Validation 2 113 Consecutive Prospective Cases		Overall Validation Process 166 Cases	
	Value	95% CI	Value	95% CI	Value	95% CI
Sensitivity	71.429%	41.896% to 91.611%	66.667%	22.278% to 95.673%	70.000%	45.721% to 88.107%
Specificity	51.282%	34.780% to 67.582%	83.178%	74.723% to 89.714%	74.658%	66.800% to 81.486%
AUC	0.614	0.470 to 0.744	0.749	0.659 to 0.826	0.723	0.649 to 0.790
Positive LR	1.466	0.924 to 2.327	3.963	1.957 to 8.024	2.762	1.852 to 4.120
Negative LR	0.557	0.230 to 1.347	0.401	0.129 to 1.247	0.402	0.204 to 0.790
Disease prevalence					0.010%	
Positive PV					0.028%	0.019% to 0.041%
Negative PV					99.996%	99.992% to 99.998%
Accuracy					74.657%	67.333% to 81.079%

LR, likelihood ratio; PV, predictive value.

**Table 5:** Reproducibility of the DSC test.

DSC Classification	FIRST TEST	At Median Follow-Up (15.5 Months)	Diagnosis	First Test	At Median Follow-Up (15.5 Months)
	n. (%)	n. (%)		n. (%)	n. (%)
negative	17 (65.4%)	15 (57.7%)	OLGA 0–II	22 (22.3%)	22 (22.3%)
neutral	3 (11.5%)	5 (19.2%)	OLGA III–IV	3 (11.5%)	3 (11.5%)
positive	6 (23.1%)	6 (23.1%)	GC	1 (3.8%)	1 (3.8%)

**Table 6:** Comparison between the predictive values of the DSC test and pepsinogen tests for GC risk.

	DSC Test		PG Test	
	Value	95% CI	Value	95% CI
Sensitivity	70.00%	45.721% to 88.107%	15.00%	3.207% to 37.893%
Specificity	74.66%	66.800% to 81.486%	78.77%	71.236% to 85.094%
AUC	0.723	0.649 to 0.790	0.470	0.391 to 0.548
Positive LR	2.762	1.852 to 4.120	0.71	0.238 to 2.099
Negative LR	0.402	0.204 to 0.790	1.08	0.881 to 1.321
Disease prevalence	0.01%		0.01%	
Positive PV	0.03%	0.019% to 0.041%	0.01%	0.002% to 0.021%
Negative PV	99.996%	99.992% to 99.998%	99.989%	99.987% to 99.991%
Accuracy	74.66%	67.333% to 81.079%	78.76%	71.748% to 84.717%

LR, likelihood ratio; PV, predictive value.

## Data Availability

The data presented in this study are available in this article.
